# REDD1 Affects Proliferation, Apoptosis, Migration, and Colony Formation via p-ERK and p-JNK Signaling in Lung Adenocarcinoma Cells Under Hypoxia

**DOI:** 10.3390/biomedicines13122918

**Published:** 2025-11-28

**Authors:** Xiaoyu Fang, Xuezhao Wang, Xiansheng Liu, Yuanzhou He

**Affiliations:** Department of Respiratory and Critical Care Medicine, Tongji Hospital, Key Lab of Pulmonary Diseases of the Health Ministry, Key Site of National Clinical Research Center for Respiratory Disease, Tongji Medical College, Huazhong University of Science and Technology, Wuhan 430000, China

**Keywords:** A549, H1299, REDD1, lung adenocarcinoma, p-ERK, p-JNK

## Abstract

**Background:** Regulated in development and DNA damage response 1 (REDD1) is a stress-related protein that is found to be involved in tumor progression. The role and internal regulatory mechanism of REDD1 in lung adenocarcinoma (LUAD) remain unidentified as of yet. **Methods**: Immunohistochemical and Western blot tests were performed to evaluate REDD1 expression in LUAD tissues. EdU staining, cell counting kit-8 assays, and colony formation analyses were conducted to estimate cell proliferation. Flow cytometry was applied to examine apoptosis, while migration was detected by a transwell assay. **Results**: REDD1 was upregulated in LUAD tissues, and hypoxia promoted the expression of REDD1 in LUAD cells. In addition, knockdown of REDD1 inhibited the increase in proliferation, migration, and colony formation induced by hypoxia in LUAD cells. Apoptosis was decreased by hypoxia and restored after REDD1 downregulation. Furthermore, p-ERK and p-JNK signaling pathways were involved in the changes in proliferation, apoptosis, and migration of LUAD cells following REDD1 knockdown under hypoxia. **Conclusions**: REDD1 may be a possible therapeutic target for LUAD.

## 1. Introduction

Non-small cell lung cancer (NSCLC) accounts for approximately 80% of lung cancers and remains the main cause of cancer-related mortality worldwide [1]. Lung adenocarcinoma (LAUD), which develops from small airway epithelial and type II alveolar cells secreting mucus and other substances, is the most common subtype of NSCLC [2]. Despite the development of targeted molecular therapy and immunotherapy, the 5-year survival rate of lung cancer is still <20% [3,4]. Therefore, more specific molecular targets should be continuously detected to prompt the advancement of precision therapies.

Genetic alterations in tumor cells lead to uncontrolled growth and a resistance to apoptosis, inducing angiogenesis, eventually resulting in metastasis [5,6]. Compared with the surrounding normal tissues, the organization of solid tumor blood vessels is stochastic, lacking a basic vascular structure, leading to a hypoxic microenvironment [7]. Previous studies suggest that hypoxia correlates with poor survival outcomes [8,9], malignancies [10], and therapy resistance in tumors [11,12]. Consequently, the investigation of tumor mechanisms in hypoxic conditions is more consistent with clinical contexts.

Regulated in development and DNA damage response 1 (REDD1), also known as “DNA damage-inducible transcript 4 (DDIT4)”, was initially discovered as the response gene of hypoxia-inducible factor 1 (HIF-1) [13]. REDD1 is reported to respond to various stresses such as endurance exercise [14], ionizing radiation [15], DNA damage, and hypoxia [16]. Recently, it has been proposed that REDD1 may be involved in numerous cancers. A previous study demonstrated that REDD1 promotes ovarian cancer metastasis by inducing cell migration and invasion [17]. Additionally, REDD1 regulates cell proliferation, apoptosis, and autophagy in bladder urothelial carcinoma cells, leading to an increase in paclitaxel resistance in bladder urothelial carcinoma cells [18]. A study based on online datasets indicated that high REDD1 expression was significantly associated with a worse prognosis in acute myeloid leukemia, breast cancer, glioblastoma multiforme, and colon, skin, and lung cancer [19]. Constitutive overexpression of REDD1 in H1299 cells has also been shown to lead to HSP27 and HSP70 induction, which are important for lung cancer cell survival and resistance to ionizing radiation [20]. In contrast, a study of human breast cancer cells indicated that melatonin enhances arsenic trioxide-induced apoptotic cell death via the sustained upregulation of REDD1 expression [21]. However, the participation of REDD1 in the progression of LUAD, especially in hypoxia, is barely understood. Thus, a better understanding of REDD1 under hypoxic conditions in LUAD tumorigenesis is crucial for the advancement of therapies for LUAD patients.

In the present study, we found that REDD1 regulates proliferation, apoptosis, migration, and colony formation via p-ERK and p-JNK signaling in LUAD cells under hypoxia. Therefore, based on our results, REDD1 may be a potentially effective therapeutic strategy for LUAD.

## 2. Materials and Methods

### 2.1. Tissues and Cell Lines

Four pairs of human LUAD and normal tissue samples and paraffin-embedded samples were collected from the Tongji Hospital of Huazhong University of Science and Technology (Wuhan, China). For the use of human samples for research purposes, approval was obtained from the ethics committee of Tongji Hospital, Tongji Medical College (project identification code, 81700051; approval date, 17 January 2022). The A549 cell line was purchased from the American Type Culture Collection (ATCC), and H1299 was obtained from the Institute of Biochemistry and Cell Biology of the Chinese Academy of Sciences (Shanghai, China). The cells were cultured in RPMI-1640 (Gibco 12633020, Waltham, MA, USA) medium supplemented with 10% fetal bovine serum (FBS) at 37 °C under a humidified atmosphere of 5% CO_2_ and exposed to an environment with 21% or 2% O_2_ in an incubator chamber (GalaxyR, RS Biotech, Alloa, UK). In vitro exposure to hypoxia was achieved by placing cells in an airtight chamber (Billups-Rothenberg, Del Mar, CA, USA) gassed with 1% O_2_, 5% CO_2_, and 94% N_2_. The chamber and the normoxic control flasks were placed in an incubator (Billups-Rothenberg, Del Mar, CA, USA) at 37 °C.

### 2.2. Regents and Antibodies

Antibodies that recognize REDD1 (10638-1-AP), matrix metallopeptidase 1 (MMP1) (10371-2-AP), matrix metallopeptidase 9 (MMP9) (60600-1-Ig), and B-cell lymphoma-2-associated X (Bax) (50599-2-Ig) were purchased from Proteintech (Wuhan, China). B-cell lymphoma-2 (Bcl2) (A00040-1) antibody was obtained from Boster (Wuhan, China). p-p44/42 MAPK (ERK1/2) (Thr202/Tyr204) (#9101), p44/42 MAPK (ERK1/2) (#4695), p-SAPK/JNK (Thr183/Tyr185) (#4668), SAPK/JNK (#9252), p-p38 MAPK (Thr180/Tyr182) (#9211), p38 mitogen-activated protein kinase (MAPK) (#9212), p-Akt (Ser473), Akt (#9271), p-mTOR (Ser2448) (#5536), and mTOR antibodies (#2974) were acquired from Cell Signaling Technology Inc. (Beverly, MA, USA), while the β-actin (DKM9001L) antibody was obtained from Sungene Biotech Co., Ltd. (Tianjin, China). The p38 and SB203580 inhibitors, Honokiol and Anisomycin, were purchased from MedChem Express LLC (Monmouth Junction, NJ, USA).

### 2.3. Immunocytochemistry

Paraffin-embedded tissues were sectioned into 4 μm slices. Sections were dewaxed and rehydrated. Endogenous peroxidase activity was blocked by treatment with 0.3% H_2_O_2_ (ServiceBio, Wuhan, China). ServiceBioSections were incubated overnight at 4 °C with anti-REDD1 antibody (10638-1-AP, 1:100, Proteintech). This was followed by incubation with peroxidase-labeled streptavidin (Chemical book, Wuahn, China, CB911980110) for 30 min at room temperature. Color development was performed using 3,3′-diaminobenzidine (DAB) (ServiceBio, Wuhan, China). Finally, sections were counterstained with hematoxylin (ServiceBio, Wuhan, China).

### 2.4. Cell Transfection

Small interfering RNA (siRNA) that targets REDD1 (si-REDD1) and nontargeting negative control siRNA (si-NC) were purchased from RiboBio (Guangzhou, China). REDD1 (50 nM) and si-NC (50 nM) were transfected into cells using Lipofectamine 3000 reagent (Invitrogen, Carlsbad, CA, USA). The medium was replaced with a new RPMI-1640 after 24 h. The sequence of si-NC was AACACCGAACGAGACAATT, while the sequence of si-REDD1 was AGACACGGCTTACCTGGAT.

For REDD1 overexpression, after transfected cells reached 90% confluence, they were cultured in serum-free medium for 2 h, then infected with either empty adenovirus (Ad-control) or adenovirus expressing REDD1 (Ad-REDD1) for 6 h. Subsequently, the cells were cultured in fresh DMEM medium (Thermo Fisher Scientific, Waltham, MA, USA) containing 10% fetal bovine serum (Thermo Fisher Scientific, Waltham, MA, USA) for 2 d. The overexpression efficiency was analyzed by PCR and Western blot.

### 2.5. Cell Proliferation Assay

Cells were seeded in 96-well plates with a density of 3000 cells per well. After transfection with siRNA or control, proliferation of cells was determined using the 5-ethynyl-2′-deoxyuridine (EdU) assay (Cell-Light™ EdU Apollo^®^643 in vitro imaging kit, RiboBio, Guangzhou, China), Hoechst 33,342 (Biyuntian Wuan, C1029), and the cell counting kit-8 (CCK-8) (Dojindo Laboratory, Tokyo, Japan) according to the manufacturer’s instructions. Absorbance at 450 nm was measured by the ELx800 Universal Microplate Reader (Bio-Tek Instruments, Winooski, VT, USA). After EdU staining, the plates were observed under a fluorescence microscope (BX53, Olympus, Hachioji, Tokyo, Japan).

### 2.6. Cell Apoptosis Assay

Cells transfected with siRNA or control were seeded in six-well plates. Cells were exposed to hypoxic conditions for 24 h and harvested by centrifugation. Apoptosis assay was performed by flow cytometry using the Annexin V-fluorescein isothiocyanate (FITC)/propidium iodide (PI) staining kit (KeyGEN, Nanjing, China).

### 2.7. Cell Migration Assay

Transwell chambers (Corning Inc., Corning, NY, USA) were coated with or without Matrigel (3384, Thermo Fisher Scientific Waltham, MA, USA) to assess cell invasion and migration, respectively. The bottom chambers contained medium with 20% FBS (ServiceBio, Wuhan, China), while the upper chambers were seeded with cells in serum-free medium. After the incubation at 37 °C for 48 h, the cells were fixed with 4% paraformaldehyde (ServiceBio, Wuhan, China), stained with crystal violet (ServiceBio, Wuhan, China), and examined under a microscope (BX53, Olympus, Hachioji, Tokyo, Japan). The transwell assay was performed using inserts with a membrane pore size of 8.0 µm.

### 2.8. Colony Formation Assay

Transfected cells were seeded in six-well plates (400 cells/well). Fourteen days later, the cells were fixed, stained, and counted. The cell colonies were fixed with 4% paraformaldehyde, stained with 0.1% crystal violet, and then manually counted. The counting was performed by two independent investigators using an inverted microscope (IX73, Olympus, Hachioji, Tokyo, Japan). Only colonies containing more than 50 cells were considered a viable colony and counted.

### 2.9. Western Blot Analysis

Total protein extracts were prepared from human lung tissues and cultured cells in a radioimmunoprecipitation assay (RIPA) lysis buffer (ServiceBio, Wuhan, China). Proteins were electrophoresed through polyacrylamide gel electrophoresis (SDS-PAGE) (ServiceBio, Wuhan, China) and transferred to 0.22 µm microporous polyvinylidene difluoride (PVDF) membranes (Roche Diagnostics, Mannheim, Germany). The membranes were blocked with 5% nonfat milk (ServiceBio, Wuhan, China) and incubated overnight at 4 °C with primary antibodies, REDD1 (1:1000), MMP1 (1:1000), MMP9 (1:1000), Bax (1:1000), Bcl2 (1:1000), p-p44/42 MAPK (ERK1/2) (1:2000), p44/42 MAPK (ERK1/2) (1:200), p-SAPK/JNK (1:1000), SAPK/JNK, p-p38 MAPK (1:1000), p38 MAPK (1:1000), p-Akt (1:1000), Akt (1:1000), p-mTOR (1:1000), and mTOR (1:1000). A total of 20–30 μg of protein per sample was loaded for SDS-PAGE. β-actin was used as the housekeeping protein for normalization. The secondary antibody used was a horseradish peroxidase (HRP)-conjugated anti-rabbit IgG (SA00001-1, Proteintech). The bands were detected using a ChemiDoc detection system (Bio-Rad, Hercules, CA, USA), and the results were analyzed using ImageJ software (v1.46r) (WS Rasband, ImageJ, NIH, Bethesda, MD, USA).

### 2.10. Statistical Analysis

Data are presented as the mean ± standard error of the mean and analyzed using GraphPad Prism software (version 8.0, San Diego, CA, USA). The significance of differences between multiple groups was assessed using a two-way analysis of variance. *p* < 0.05 was considered statistically significant.

## 3. Results

### 3.1. REDD1 Is Upregulated in Lung Adenocarcinoma

**Figure 1:**
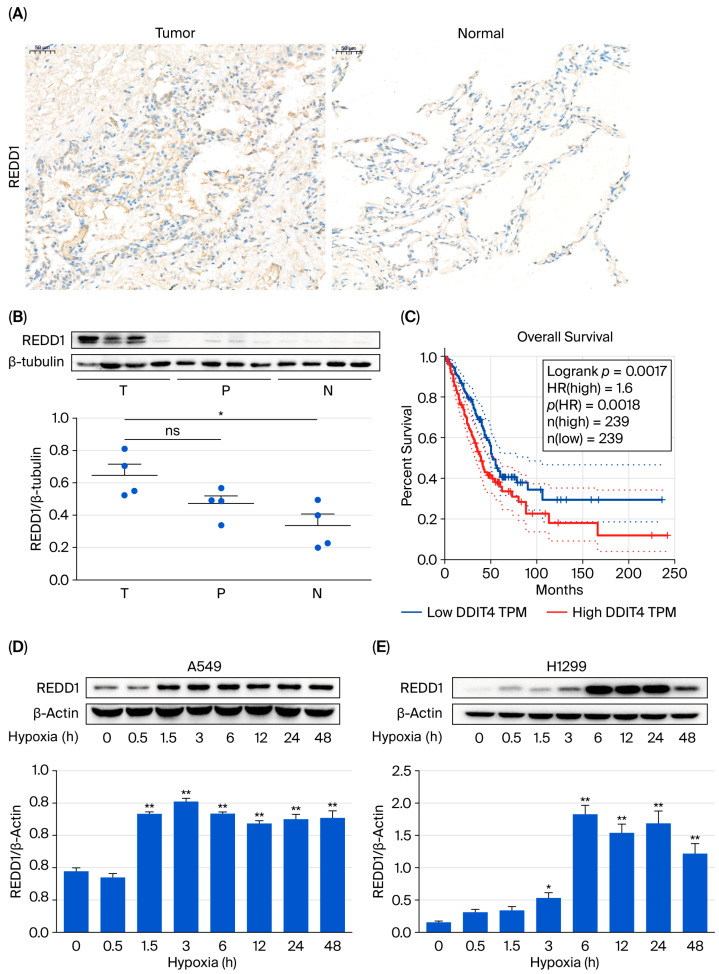
REDD1 is overexpressed in LUAD. (**A**) Immunohistochemistry staining images (200X) of REDD1 expression in four pairs of LUAD and normal tissues. (**B**) Western blot analyses of REDD1 protein level in samples of four matched LUAD (T), para-carcinoma tissues (P), and normal tissues (N); β-tubulin was used as the control. (**C**) Patients with high REDD1 expression exhibit a significantly reduced overall survival compared to those with low REDD1 expression. (Logrank p, *p*-value from log-rank test; HR, hazard ratio; p (HR), survival probability; n (high), number of high REDD1 expression; n (low), number of low REDD1 expression; TPM, transcripts per million). (**D**,**E**) Protein expression of REDD1 in A549 and H1299 cells at different durations of hypoxia. E, *n* = 4; F, *n* = 6. * *p* < 0.05; ** *p* < 0.01.

Immunohistochemistry for REDD1 expression was performed in paraffin sections from four pairs of LUAD and normal tissue samples. Furthermore, Western blots were carried out on four matched LUAD tissues (T), para-carcinoma tissues (P), and normal tissues (N), while β-tubulin was used as the control. REDD1 was highly overexpressed in the tumor tissue compared with the matched normal tissue (Figure 1A,B). In addition, interactive analysis of the gene expression profiles of 239 LUAD patients revealed that high REDD1 expression was correlated with poor overall survival (Figure 1C). As hypoxia is a driving force of tumor progression, REDD1 protein expression was detected in A549 and H1299 cells at different hypoxia times. The results showed that the expression of REDD1 increases in a hypoxic-time-dependent manner in both A549 and H1299 cells (Figure 1D,E). In addition, gene expression profiling interactive analysis (GEPIA) 2.0 showed the difference in the REDD1 expression in LUAD and normal tissues (Figure S7A). Furthermore, REDD1 expression was significantly correlated with the pathological stage of tumors in LUDA (Figure S7B).

### 3.2. REDD1 Downregulation Suppresses Proliferation of LUAD Cells Under Hypoxia

**Figure 2:**
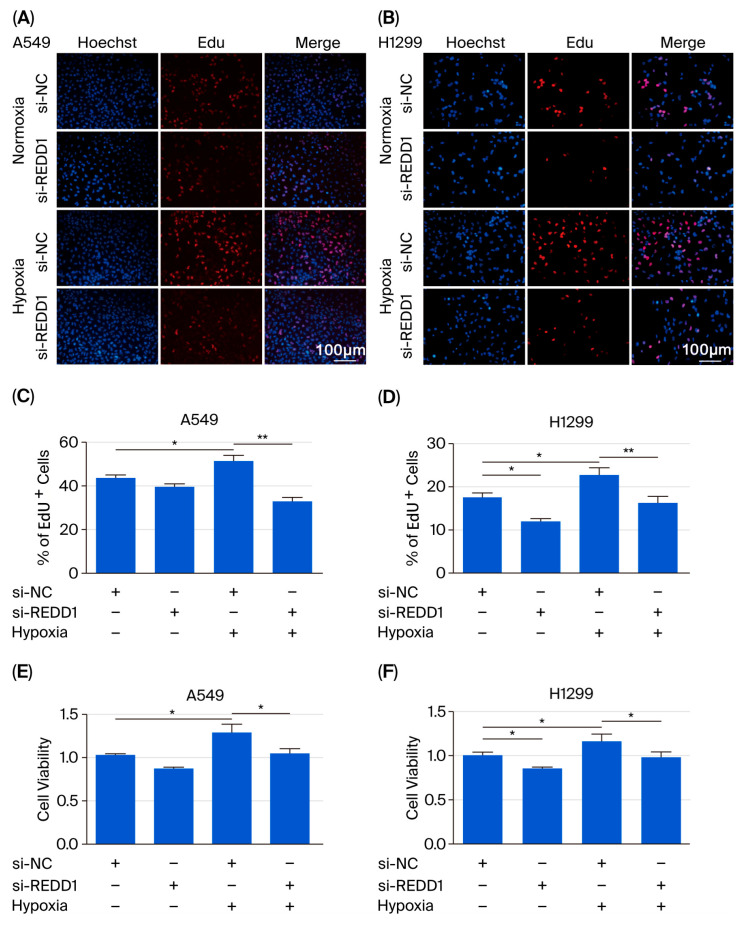
Knockdown of REDD1 inhibits A549 and H1299 cell proliferation under hypoxia. (**A**,**B**) A549 and H1299 cells were transfected with si-REDD1 under normoxic and hypoxic conditions. Then, the EdU assay was applied. (**C**,**D**) Quantitation of EdU^+^ cells. (**E**,**F**) Cell viability was determined using the CCK-8 assay under the same conditions as in A. *n* = 4. Scale bar, 100 μm. * *p* < 0.05; ** *p* < 0.01.

To investigate the impact of REDD1 on lung adenocarcinoma progression, we used siRNA to downregulate REDD1 expression in LUAD cells. Prior to describing the results, the experimental procedure is outlined as follows: A549 and H1299 cells transfected with si-REDD1 were cultured under normoxic and hypoxic conditions following 24 h of hypoxia treatment, after which the cells were collected. Our findings indicate that REDD1 exhibits significantly elevated expression in LUAD cells under hypoxic conditions, and this upregulation is markedly suppressed upon si-REDD1 inhibition (Figure S1A,B). Additionally, we have evaluated the efficiency of REDD1 overexpression (Figure S1C,D). The EdU analysis revealed that proliferation of cells was upregulated in hypoxic conditions compared to normoxic conditions and was downregulated after REDD1 knockdown (Figure 2A–D). In addition, cell viability was assessed using the CCK-8 assay. As presented in Figure 2E,F, hypoxia increased cell viability, while transfection with si-REDD1 could attenuate this increase.

### 3.3. Silencing of REDD1 Promotes A549 and H1299 Cell Apoptosis Under Hypoxia

**Figure 3:**
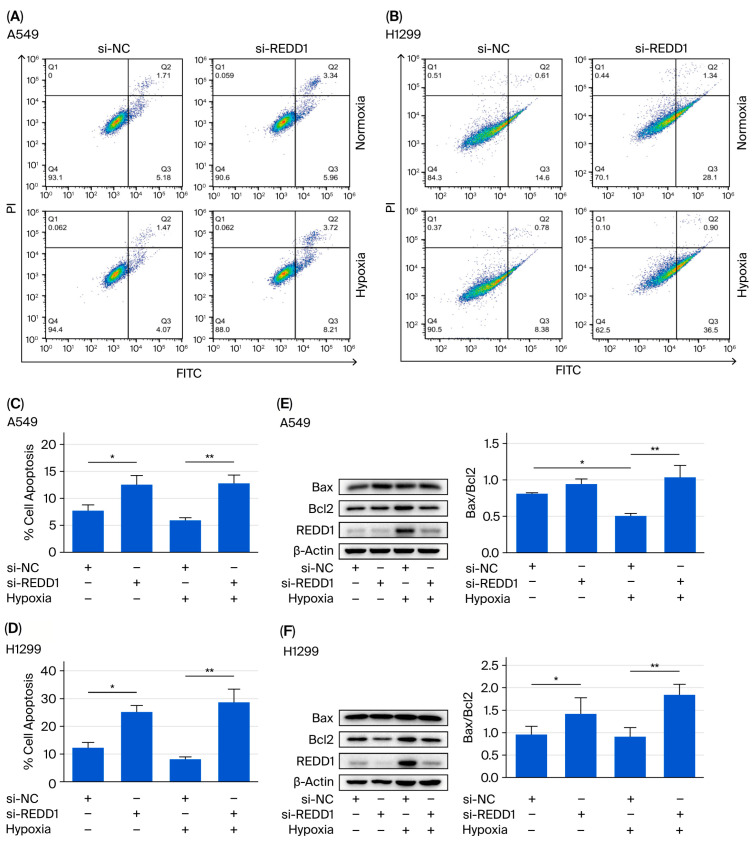
Downregulation of REDD1 promotes A549 and H1299 cell apoptosis under hypoxia. (**A**,**B**) Apoptosis of A549 and H1299 cells following the knockdown of REDD1 was detected by flow cytometric analysis of Annexin V-FITC/PI. (**C**,**D**) Quantitation of apoptotic cells. (**E**,**F**) Protein expression of Bax and Bcl2 was determined by Western blot. *n* = 4. * *p* < 0.05; ** *p* < 0.01.

To confirm the effect of REDD1 on apoptosis, Annexin-V-FITC/PI staining was performed in A549 and H1299 cells. While hypoxia alone did not cause a statistically significant decrease in apoptosis, the trend toward decreased apoptosis under hypoxia was significantly reversed by REDD1 depletion (Figure 3A–D). Moreover, the Bax/Bcl2 ratio, an indicator of apoptosis, showed a decreasing trend after hypoxia in H1299 cells, although this change was not statistically significant. However, REDD1 silencing significantly upregulated the Bax/Bcl2 ratio under hypoxic conditions (Figure 3E,F).

### 3.4. Cell Migration and Colony Formation Are Suppressed After REDD1 Knockdown Under Hypoxia

**Figure 4:**
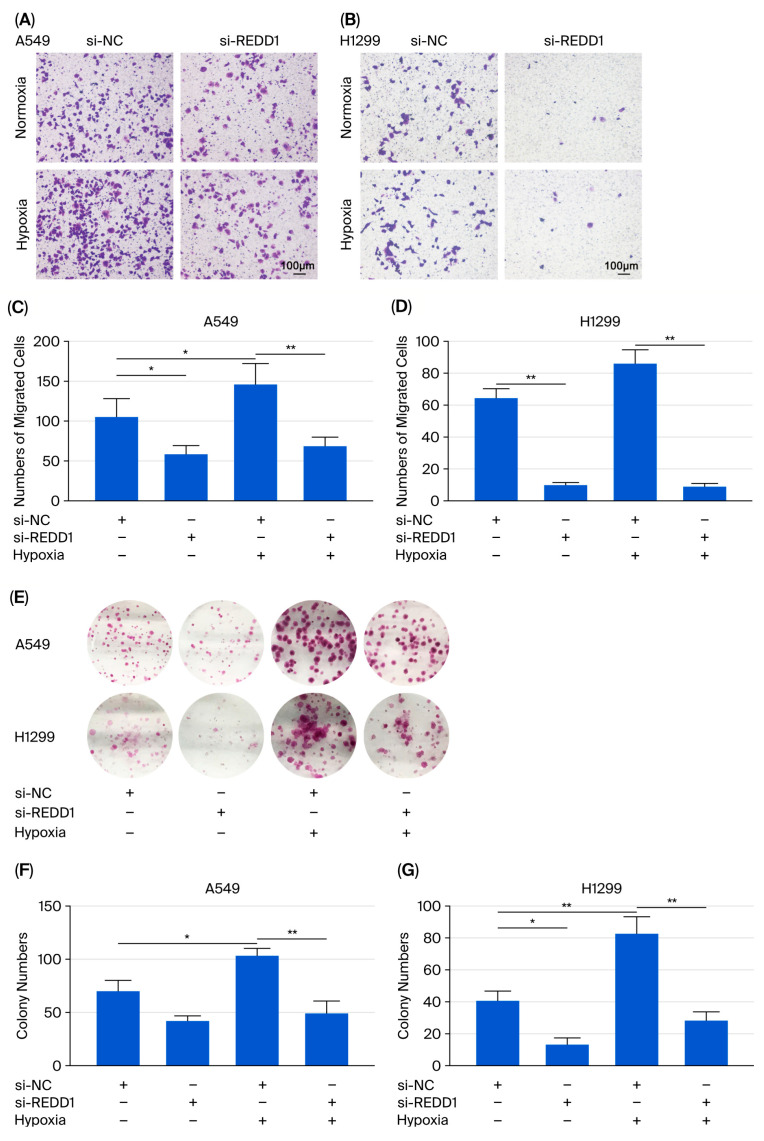
Cell migration and colony formation were suppressed after REDD1 knockdown under hypoxia. (**A**,**B**) Images of crystal violet staining. (**C**,**D**) The number of migrating cells was analyzed. (**E**–**G**) Colony formation assay was performed in A549 and H1299 cells. *n* = 4. Scale bar, 100 μm. * *p* < 0.05; ** *p* < 0.01.

An increase in A549 and H1299 cell migration was observed under hypoxic conditions, and the REDD1 knockdown reversed this increase (Figure 4A,B,D,E). REDD1 knockdown significantly reduced the colony formation of the two lung adenocarcinoma cell lines under hypoxia (Figure 4C,F,G). Furthermore, there was a significant downregulation of the expression of MMP9 upon REDD1 knockdown, while any difference in MMP1 expression was not observed (Figure S2A,B).

### 3.5. Variation in Signaling Cascade Protein Expression After REDD1 Knockdown

**Figure 5:**
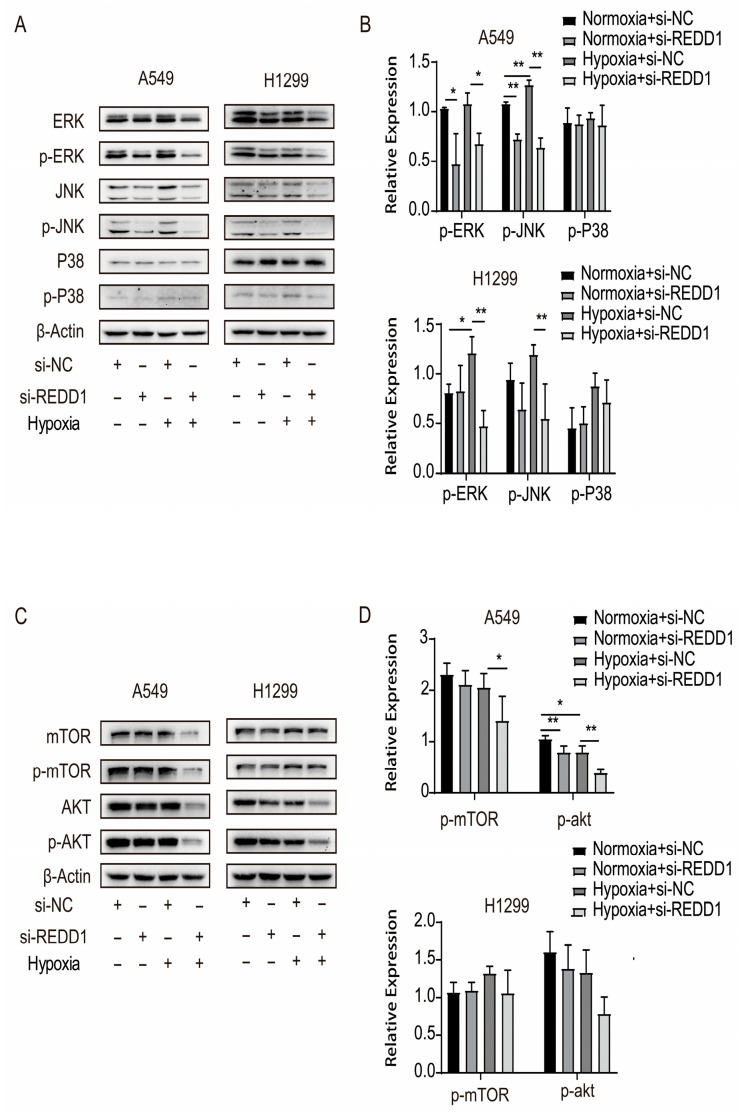
Signaling pathway protein expression after REDD1 knockdown. (**A**,**B**) Western blot results for MAPK signaling proteins p-ERK, p-JNK, and p-p38 MAPK are shown in A549 and H1299 cells. (**C**,**D**) Identification of p-AKT and p-mTOR in A549 and H1299 cells. *n* = 4. * *p* < 0.05; ** *p* < 0.01.

To explore the mechanism of REDD1 signaling involvement in LUAD, Western blots were utilized to assess the expression of relevant signaling proteins. We examined the MAPK family (ERK1/2, JNK, and p38 MAPK) and mTOR and Akt signaling pathways and revealed that phosphorylation of ERK1/2 and JNK was notably increased in A549 and H1299 under hypoxia and decreased after transfection with si-REDD1 (Figure 5A,B). On the contrary, phosphorylation of P38 did not increase in hypoxic A549 or H1299 cells (Figure 5A,B). Similarly, hypoxia did not increase phosphorylation of mTOR in either A549 or H1299 cells (Figure 5C,D).

In contrast to Akt, the phosphorylation level of mTOR in both A549 and H1299 cells remained unchanged upon hypoxia treatment. Furthermore, knocking down REDD1 under hypoxia did not elicit a significant change in mTOR phosphorylation (Figure 5C,D), indicating that mTOR activity is not regulated by the REDD1-Akt axis under these experimental conditions.

Moreover, to evaluate how REDD1 might regulate ERK and JNK phosphorylation, we resorted to using LinkedOmics (http://www.linkedomics.org/login.php (accessed on 1 October 2025) (Figure S7C,D). The results demonstrated that REDD1 is closely related to MAPK and may regulate downstream ERK and JNK phosphorylation.

### 3.6. REDD1 Regulates LUAD Cell Proliferation, Apoptosis, Migration, and Colony Formation Via p-ERK and p-JNK Signaling Under Hypoxia

**Figure 6:**
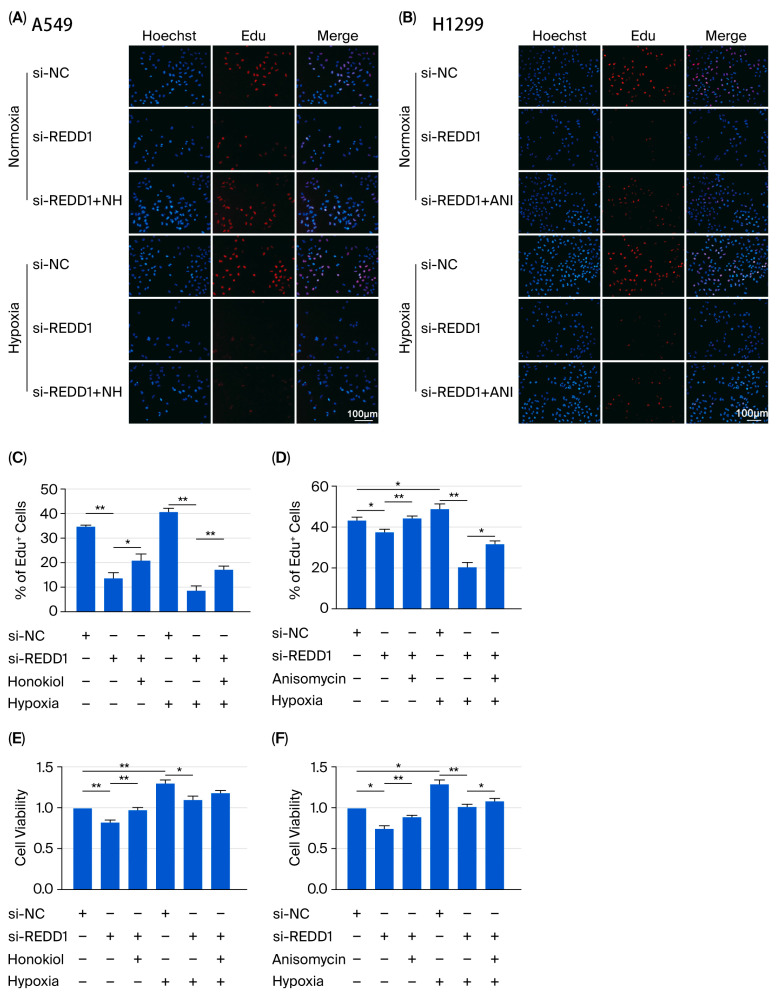

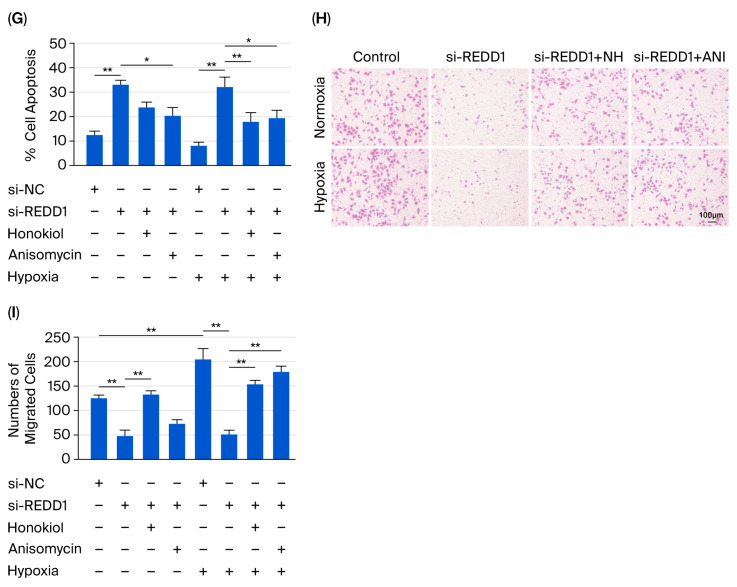
HN and ANI reverse the impact of the A549 cell line behavior. Cells were transfected with si-REDD1. (**A**–**D**) The EdU assay was used to measure cell proliferation. (**E**,**F**) Cell viability was determined by the CCK-8 test. (**G**) Annexin V-FITC/PI staining was used to evaluate cell apoptosis. (**H**,**I**) Images of cell migration are presented and quantified. *n* = 4. ANI, anisomycin, HN, honokiol, scale bar, 100 μm. * *p* < 0.05 and ** *p* < 0.01.

To examine the effect of ERK and JNK signaling pathways on hypoxic LUAD cells after REDD1 knockdown, a rescue experiment was performed using the activator of ERK, Honokiol (HN), and JNK, Anisomycin (ANI), in REDD1-depleted cells [22]. A549 cells were treated with HN (10 μM) or ANI (10 nM) under normoxic or hypoxic conditions following transfection with si-REDD1 and harvested after 24 h for the following tests. The effects of HN and ANI on cell viability in LUAD cells were tested (Figure S3). The results of the EdU staining (Figure 6A–D) and CCK-8 assay (Figure 6E,F) showed that ANI stimulation significantly promotes cell proliferation after REDD1 knockdown under hypoxia. A similar promotive trend was observed with HN treatment, although it did not reach statistical significance in the CCK-8 assay (Figure 6E). Furthermore, the increase in apoptosis induced by REDD1 knockdown was significantly inhibited by HN and ANI in hypoxia (Figure 6G). Moreover, the migration ability under hypoxia was reduced by REDD1 downregulation, and HN or ANI treatments could reverse this (Figure 6H). Parallel results for the H1299 cells are presented in Figure S4. Consistent findings were observed in H1299 cells. Under hypoxic conditions, the increased apoptosis induced by REDD1 knockdown was significantly suppressed by HN and ANI treatments (Figure S4A–F). Moreover, REDD1 downregulation attenuated cellular migratory capacity. This effect showed a trend toward reversal upon HN or ANI administration, although it did not reach statistical significance in the case of the migration assay (Figure S4G–I).

At the protein level, we observed that under hypoxic conditions, knockdown of REDD1 led to the inhibition of p-ERK and p-JNK in LUAD cells, and the reversal of p-JNK inhibition by Anisomycin, while observable, did not reach statistical significance in A549 cells (Figure S5B), in contrast to the significant effect observed for p-ERK (Figure S5A). Overexpression of REDD1 significantly activates the expression of p-ERK and p-JNK in LUAD cells, a phenomenon that is markedly attenuated upon the addition of p-ERK (Figure S6A) and p-JNK (Figure S6B) inhibitors. This further substantiates that REDD1 may exert its biological effects by modulating the p-ERK and p-JNK signaling pathways in a hypoxic environment. Furthermore, we used LinkedOmics to evaluate how REDD1 might regulate ERK and JNK phosphorylation. The results demonstrated that REDD1 is closely related to the MAPK signaling pathway and may upstream regulate ERK and JNK phosphorylation (Figure S7C,D).

### 3.7. REDD1 Overexpression Regulates Cell Proliferation, Apoptosis, Migration, and Colony Formation in Normoxic A549 and H1299 Cells

**Figure 7:**
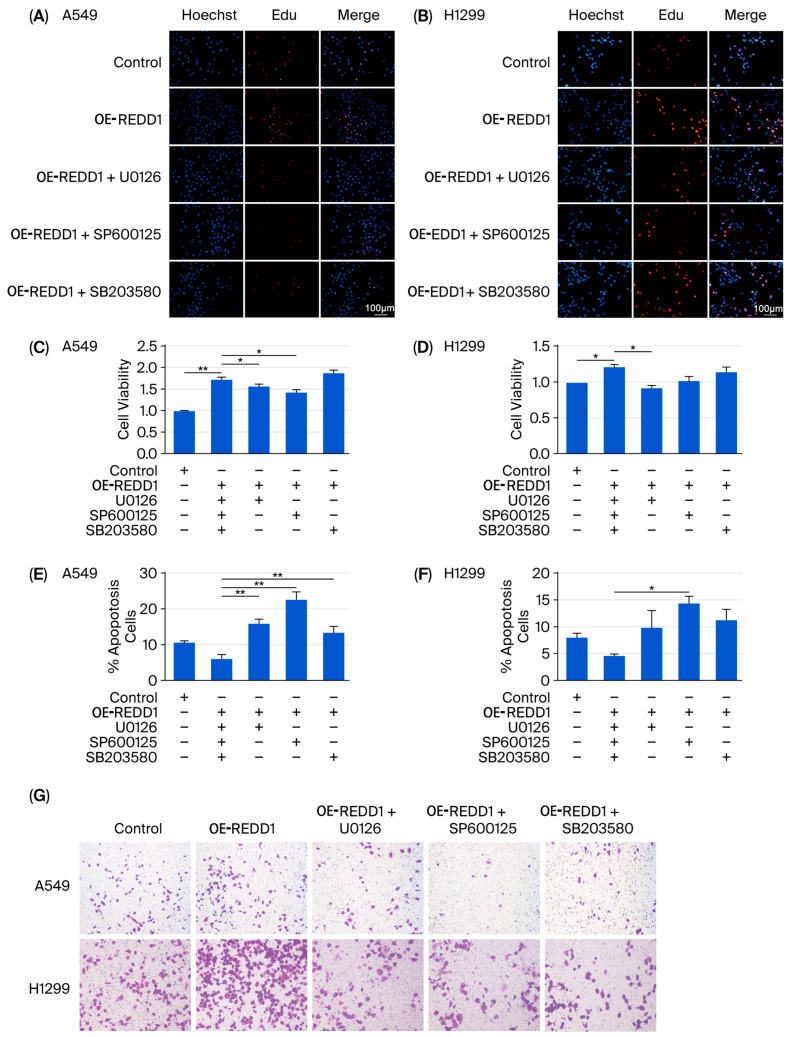
REDD1 regulates cell biological behavior in normoxic A549 and H1299 cells. Cells were transfected with REDD1 plasmid. Then, they were treated with U0126 (10 μM), SP600125 (10 μM), or SB203580 (10 μM) for 24 h under normoxia. (**A**,**B**) Cell proliferation was assessed by EdU assay. (**C**,**D**) The CCK-8 test was used to estimate cell viability. (**E**,**F**) Flow cytometry was used to detect apoptosis. (**G**) Cell migration and colony formation were presented using crystal violet staining. *n* = 4. * *p* < 0.05; ** *p* < 0.01.

To further confirm the signaling pathways in hypoxic LUAD cells, the ERK inhibitor U0126, JNK inhibitor SP600125, and p38 inhibitor SB203580 were used following REDD1 overexpression. After transfection with a REDD1 overexpression plasmid, cells were incubated with U0126 (10 μM), SP600125 (10 μM), or SB203580 (10 μM) for 24 h under normoxic conditions. The results of the EdU assay (Figure 7A,B) (this effect was observed in approximately 85% of the cells, and the signal intensity was consistently increased by more than 2-fold in the REDD1 overexpression group compared to the control) and the CCK-8 test (Figure 7C,D) indicated that REDD1 overexpression promoted cell proliferation. This effect was abolished by the inhibition of ERK, whereas the inhibition of JNK attenuated, but did not fully abolish, the proliferative effect, particularly in A549 cells. The decreased apoptosis induced by REDD1 overexpression was restored by the inhibition of ERK and JNK (Figure 7E,F) in A549 cells. The migration ability was enhanced by REDD1 overexpression, and U0126 and SP600125 counteracted the changes in both cell lines (Figure 7G).

## 4. Discussion

LUAD is the most common type of lung cancer, comprising about 40% of all cases, and the incidence of LUAD has been increasing steadily over the years [1]. Because of late-stage diagnosis and a lack of effective therapeutic targets, LUAD has one of the highest mortality rates among cancers [2]. With the appearance of epidermal growth factor receptors, tyrosine kinase inhibitors (TKIs), and immunotherapy, approaches to treating LUAD have advanced dramatically in the past few decades [23,24]. However, a considerable proportion of patients with LUAD may face major resistance challenges [25]. The urgent need for novel drugs that demonstrate better efficacy and overcome resistance has propelled research to identify potential targets.

Our study indicates that REDD1 is an essential regulator prominently upregulated. REDD1 is overexpressed in hypoxic LUAD cells compared to normoxic cells. Moreover, knockdown of REDD1 suppresses the increase in proliferation, migration, and colony formation caused by hypoxia in LUAD cells. In addition, REDD1 is involved in hypoxia-induced apoptosis. Mechanically, alterations in biological behavior may be associated with the activation of the p-ERK and p-JNK signaling pathways.

Hypoxia is an essential feature of the tumor microenvironment and contributes to malignant tumor phenotypes [26]. Tumor cells proliferate to ensure survival and avoid host immune attacks. To acclimate to hypoxia, tumor cells develop defective apoptosis through HIF1-mediated variations in pro- and anti-apoptotic members of the Bcl-2 protein family, which represents a pivotal explanation for drug resistance [27]. A recent study showed that hypoxia is a driving force for resistance to EGFR TKIs via an increased expression of the fibroblast growth factor receptor and MAPK pathway in NSCLC [12]. In this study, we found that hypoxia promoted cell proliferation, migration, and colony formation and inhibited apoptosis in LUAD cells, indicating that hypoxia is involved in LUAD progression.

REDD1, which is an HIF-1-responsive gene, may be induced by hypoxia [13]. HIF-1 activates the transcription of genes that are involved in cancer biology, including cell survival, apoptosis, and invasion, which have been associated with increased patient mortality in several cancer types [28,29]. In tumor-associated macrophages of gastric cancer, HIF-1α suppresses the expression of miR-30c and results in increased REDD1 expression [30]. In this study, we detected REDD1 protein expression in LUAD tissues and found that REDD1 was notably upregulated in LUAD tissues. REDD1 was also overexpressed in LUAD cells under hypoxic conditions. Consistent with the results, the ratio of Bax/Bcl2 was upregulated after silencing REDD1 expression under hypoxia in A549 and H1299 cells. REDD1 knockdown in hypoxic LUAD cells inhibited the expression of migration-related protein MMP9, while a difference in the MMP1 expression was not observed. We demonstrate that REDD1 promotes the tumorigenicity of LUAD under hypoxia.

It has been reported that the MAPK signaling pathway regulates growth and apoptosis in response to DNA damage [31,32]. In addition, REDD1 is required for the inhibition of the mTOR1 signaling pathway in response to stress [33]. A previous study suggested that the combination of melatonin and arsenic trioxide upregulated REDD1 expression and inhibited mTORC1, thereby activating the p38/JNK pathway in human breast cancer cells [21]. However, the specific pathway regulated by REDD1 under hypoxic conditions in LUAD remains unclear. In this study, we found that REDD1 upregulation might be associated with the activation of the phosphorylation ERK and JNK signaling pathways, resulting in subsequent alteration of proliferation, apoptosis, and migration. To further verify this hypothesis, the p-ERK activator HN and p-JNK activator ANI were used. The effects of REDD1 knockdown under hypoxia could be reversed by the p-ERK activator and p-JNK activator in A549 cells. Nevertheless, the increase in apoptosis induced by REDD1 knockdown was not significantly inhibited by HN and ANI in hypoxic H1299 cells. The explanation for the inconsistency of the A549 and H1299 cells may be the heterogeneity between different LUAD cell lines. Furthermore, we used inhibitors of the MAPK signaling pathway to stimulate the LUAD cells after REDD1 overexpression. Cell proliferation and migration under REDD1 overexpression in A549 cells were impaired by U0126 and SP600125, and the decrease in apoptosis caused by REDD1 overexpression was restored by the U0126, SP600125, and SB203580 treatment in normoxic A549 cells. Thus, we identified that the activation of phosphorylation and protein levels was regulated by REDD1 in LUAD cells under hypoxia.

To evaluate REDD1 expression level and how REDD1 might regulate ERK and JNK phosphorylation, gene expression profiling interactive analysis (GEPIA 2.0) was used. We found that the expression of REDD1 in the database showed no significant difference between tumor and normal tissues. However, REDD1 was significantly correlated with the pathological stage of tumors in LUDA. Although the analysis indicated that the expression level of REDD1 did not change significantly, which was inconsistent with our experimental results. However, the results we obtained from Western blot and immunohistochemistry were more consistent with the clinical prognosis analysis results than those from the database, which were based on gene chip and PCR results. Furthermore, we used LinkedOmics to evaluate how REDD1 might regulate ERK and JNK phosphorylation. The results demonstrated that REDD1 is closely related to the MAPK signaling pathway and may upstream regulate ERK and JNK phosphorylation.

In conclusion, our results indicated that REDD1 facilitates proliferation, migration, and colony formation and alleviates hypoxia-induced apoptosis of LUAD cells mediated by the p-ERK and p-JNK pathways, thereby promoting the oncogenicity of LUAD cells. REDD1 might be an effective therapeutic target for LUAD.

## Data Availability

The original contributions presented in this study are included in the article/Supplementary Material. Further inquiries can be directed to the corresponding author(s).

## Supplementary Materials

The following supporting information can be downloaded at https://www.mdpi.com/article/10.3390/biomedicines13122918/s1: Figure S1: The protein levels of REDD1 demonstrate the knockdown efficiency of si-RNA; Figure S2: Migration proteins are altered after REDD1 knockdown; Figure S3: Effects of HN and ANI on cell viability in A549 and H1299 cells; Figure S4: HN and ANI reverse the impact of H1299 cell biological behavior; Figure S5: Validation of the activators of ERK and JNK pathways; and Figure S6: Efficiency of the inhibitors of ERK and JNK pathways. Figure S7. Analyzing LUAD and normal REDD1 expression using the GEPIA2 database and identifying REDD1 cellular processes by GSEA enrichment method using LinkedOmics.
