# Supplementary material for: REDD1 Affects Proliferation, Apoptosis, Migration, and Colony Formation via p-ERK and p-JNK Signaling in Lung Adenocarcinoma Cells Under Hypoxia
Source: Biomedicines. 2025 Nov 28;13(12):2918. doi: 10.3390/biomedicines13122918 (PMC12731090; doi:10.3390/biomedicines13122918)
Supplement: Supplementary file 1 [file biomedicines-13-02918-s001.zip › Supplementary Figure legends.pdf]

Supplementary figure legends:

Figure S1. The protein levels of REDD1 were assayed to determine the knockdown efficiency of si-RNA in (A) A549 cells (N = 5) and (B) H1299 cells (N = 4). (C, D) Protein levels of REDD1 after transfection with an overexpression plasmid in 48h. *P < 0.05, **P < 0.01.

Figure S2. REDD1 depletion affects the expression of cell migration-related protein MMP9. (A) MMP9 and MMP1 expression were probed by Western blotting. (B) and (C) Quantitation data of A. n = 4. *P < 0.05, **P < 0.01.

Figure S3. Effects of HN and ANI on cell viability in A549 and H1299 cells. n = 4.

Figure S4. HN and ANI reverse the impact of H1299 cell biological behavior. (A) Cells were transfected with si-REDD1, then they were treated with HN (10 μM) or ANI (10 nM) under normoxia or hypoxia. (A–D) EdU assay were used to measure cell proliferation. (E, F) Cell viability was determined by the CCK-8 assay. (G) Annexin V/PI staining was used to evaluate cell apoptosis. (H, I) Images of cell migration are depicted. ANI, Anisomycin; HN, Honokiol. Scale bar, 100 μm. *P < 0.05, **P < 0.01.

Figure S5. Validation of the activators of the ERK and JNK pathways. (A) The effects of HN (10 μM) in A549 and H1299 cells, n = 3. (B) The effects of ANI (10 nM) in A549 and H1299 cells. n = 5. HN, Honokiol; ANI, anisomycin. *P < 0.05, **P < 0.01.

Figure S6. Efficiency of the inhibitors of the ERK and JNK pathways. (A) The influence of U0126 (10 μM) in A549 and H1299 cells. n = 4. ANI, Anisomycin; HN, Honokiol. *P < 0.05, **P < 0.01.

Figure S7. Analyzing LUAD and normal REDD1 expression using the GEPIA2 database and identifying REDD1 cellular processes by GSEA enrichment method using LinkedOmics. (A) GEPIA 2.0 showed the difference in REDD1 expression in LUAD and normal tissues. (B) GEPIA 2.0 found that REDD1 was significantly correlated with the pathological stage of tumors in Lung adenocarcinoma (LAUD). (C, D) Evaluate the relationship of REDD1 and ERK and JNK phosphorylation. (Logrank p, p-value from Log-rank test; HR, Hazard Ratio; TPM,Transcripts Per Million, FDR, false discovery rate).
