# Supplementary figures and images for: REDD1 Affects Proliferation, Apoptosis, Migration, and Colony Formation via p-ERK and p-JNK Signaling in Lung Adenocarcinoma Cells Under Hypoxia
Source: Biomedicines. 2025 Nov 28;13(12):2918. doi: 10.3390/biomedicines13122918 (PMC12731090; doi:10.3390/biomedicines13122918)

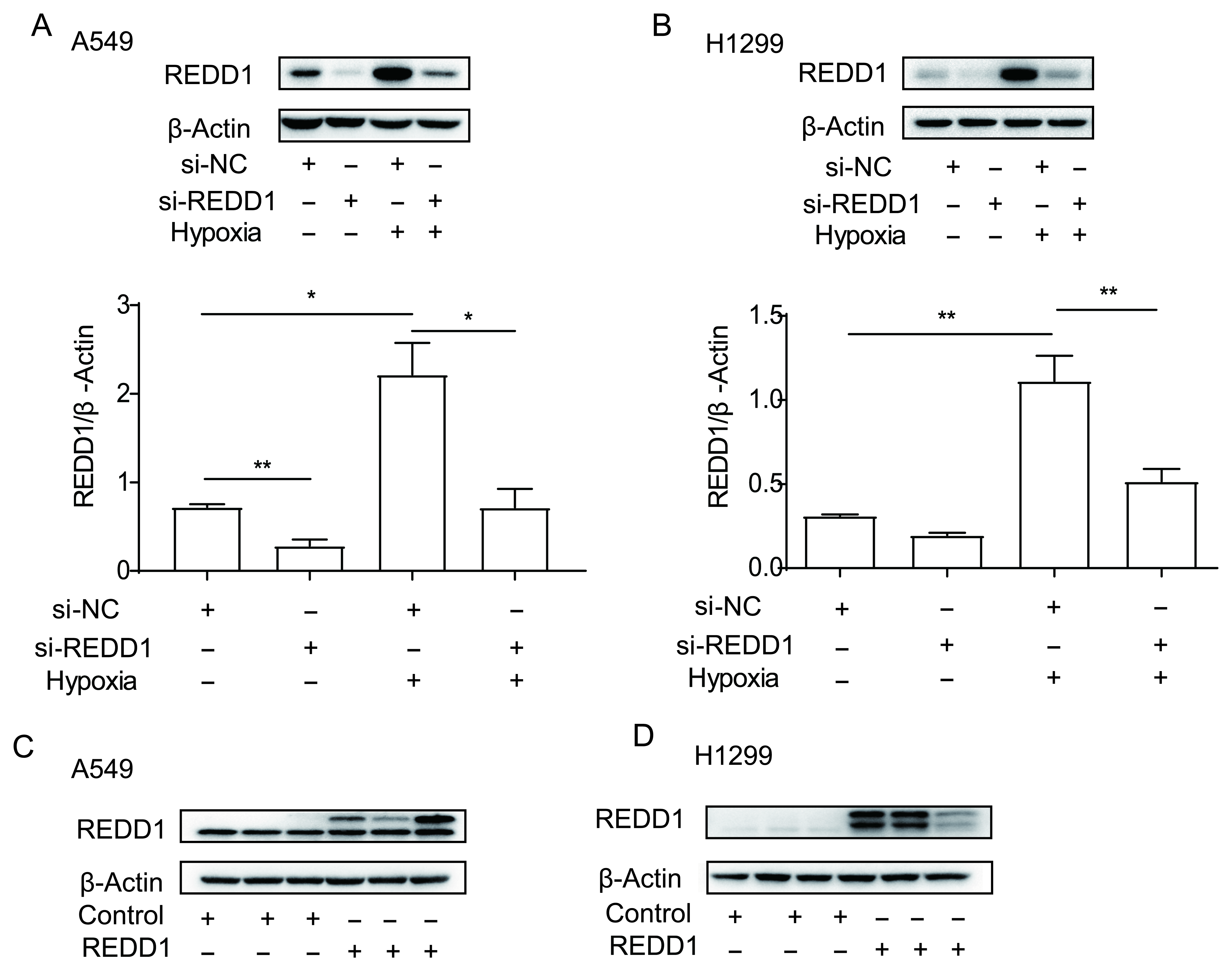

Supplement: Supplementary file 1 [file biomedicines-13-02918-s001.zip › Figure S1.tif]

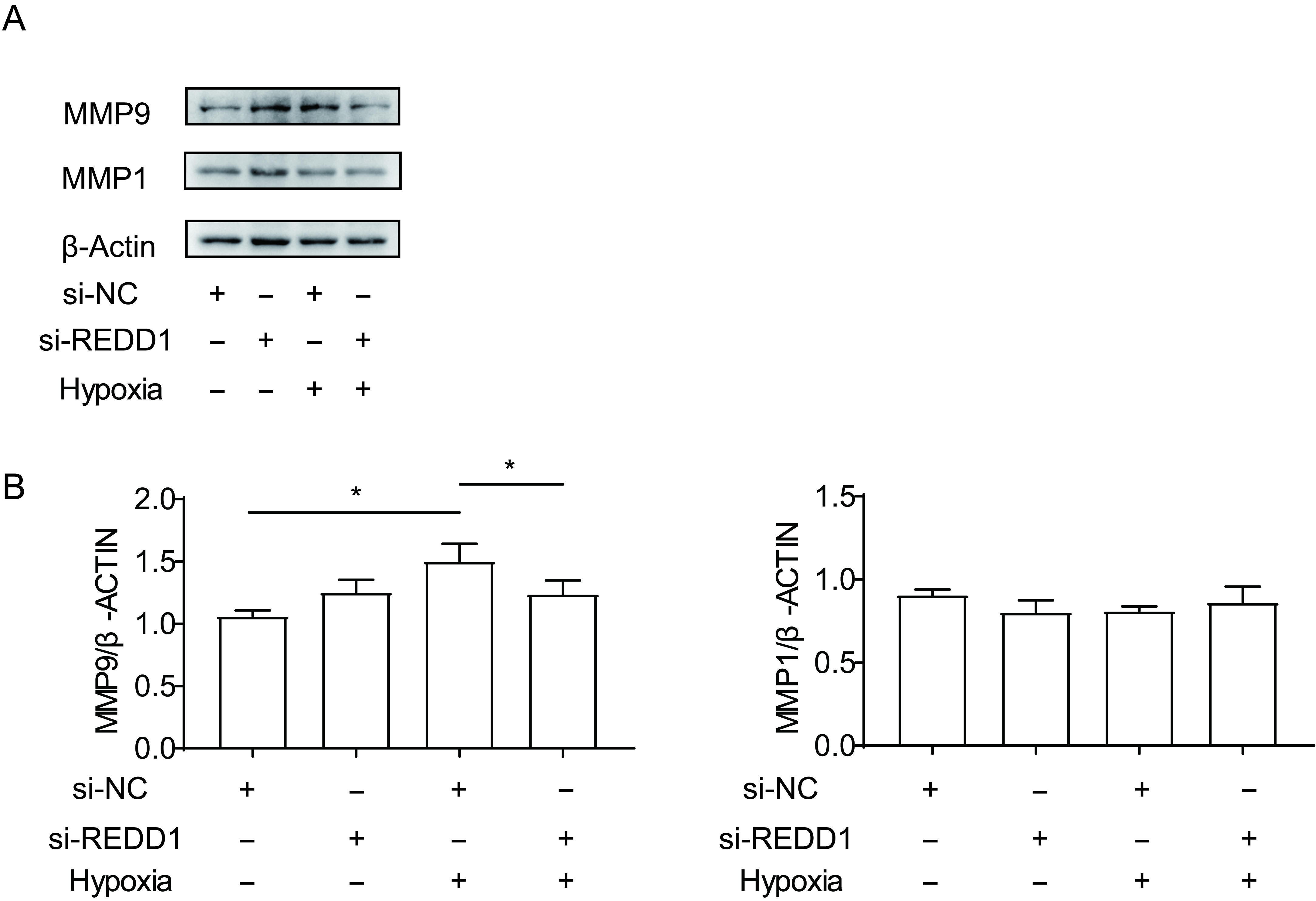

Supplement: Supplementary file 1 [file biomedicines-13-02918-s001.zip › Figure S2.tif]

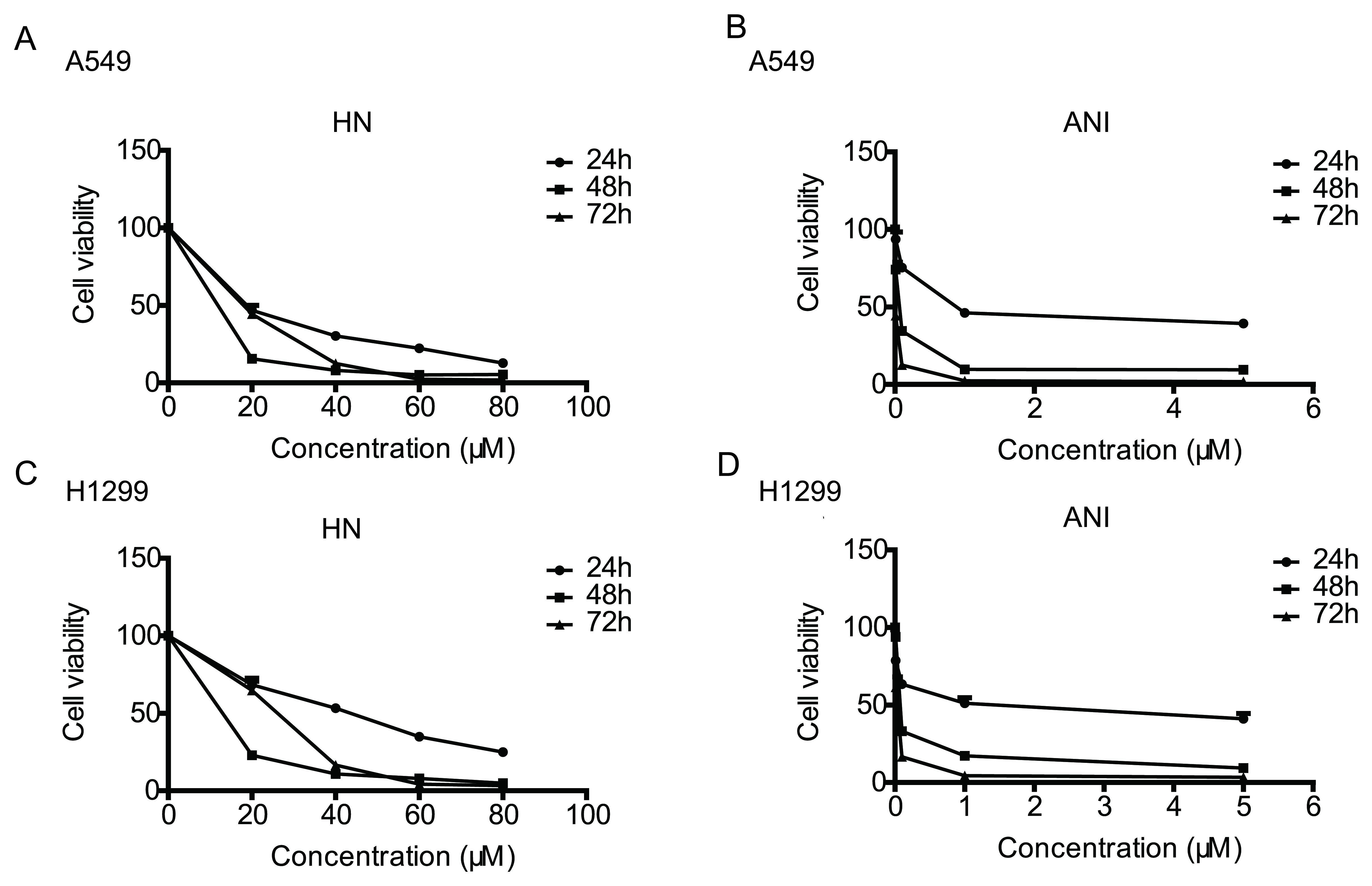

Supplement: Supplementary file 1 [file biomedicines-13-02918-s001.zip › Figure S3.tif]

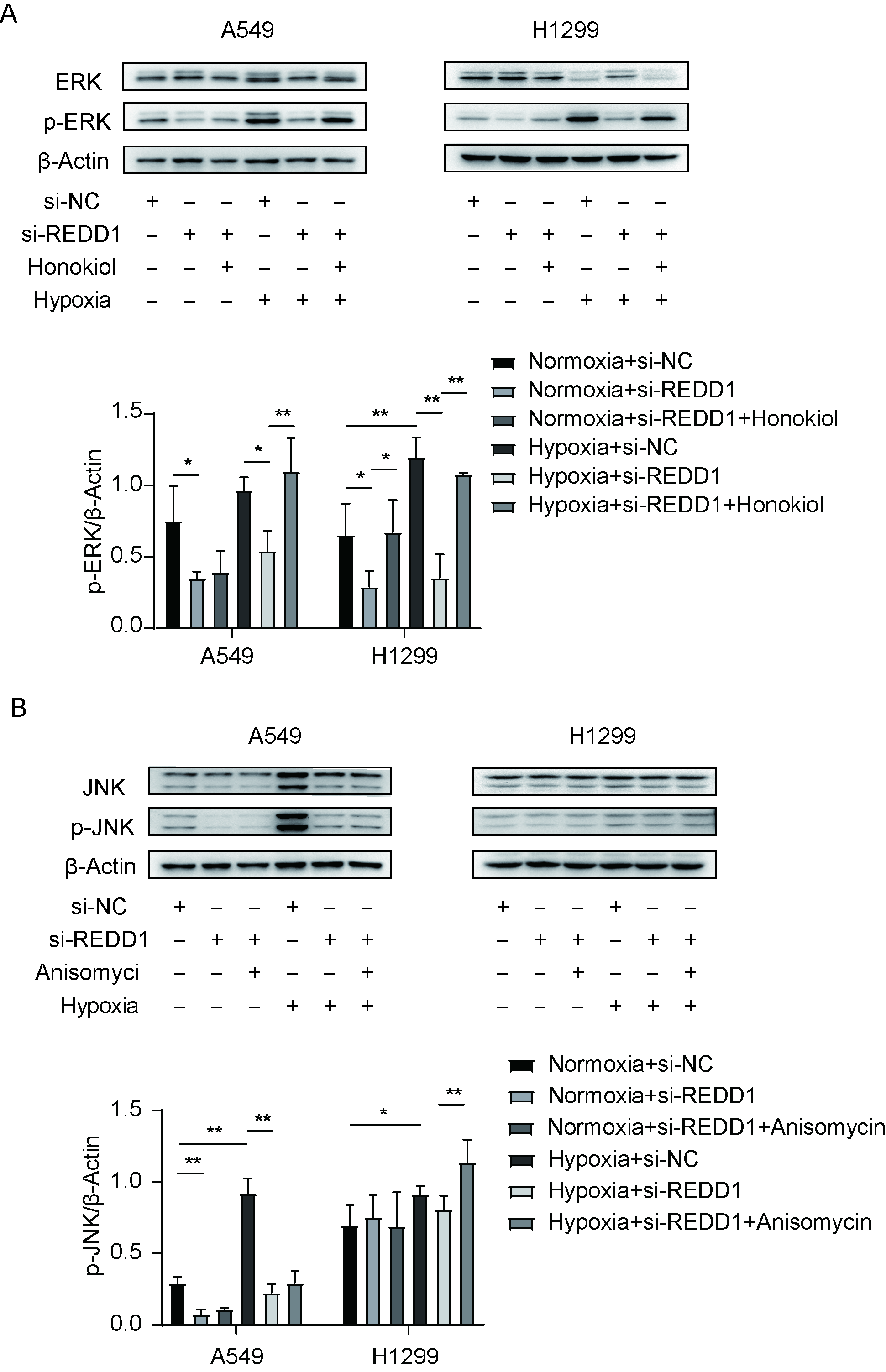

Supplement: Supplementary file 1 [file biomedicines-13-02918-s001.zip › Figure S5.tif]

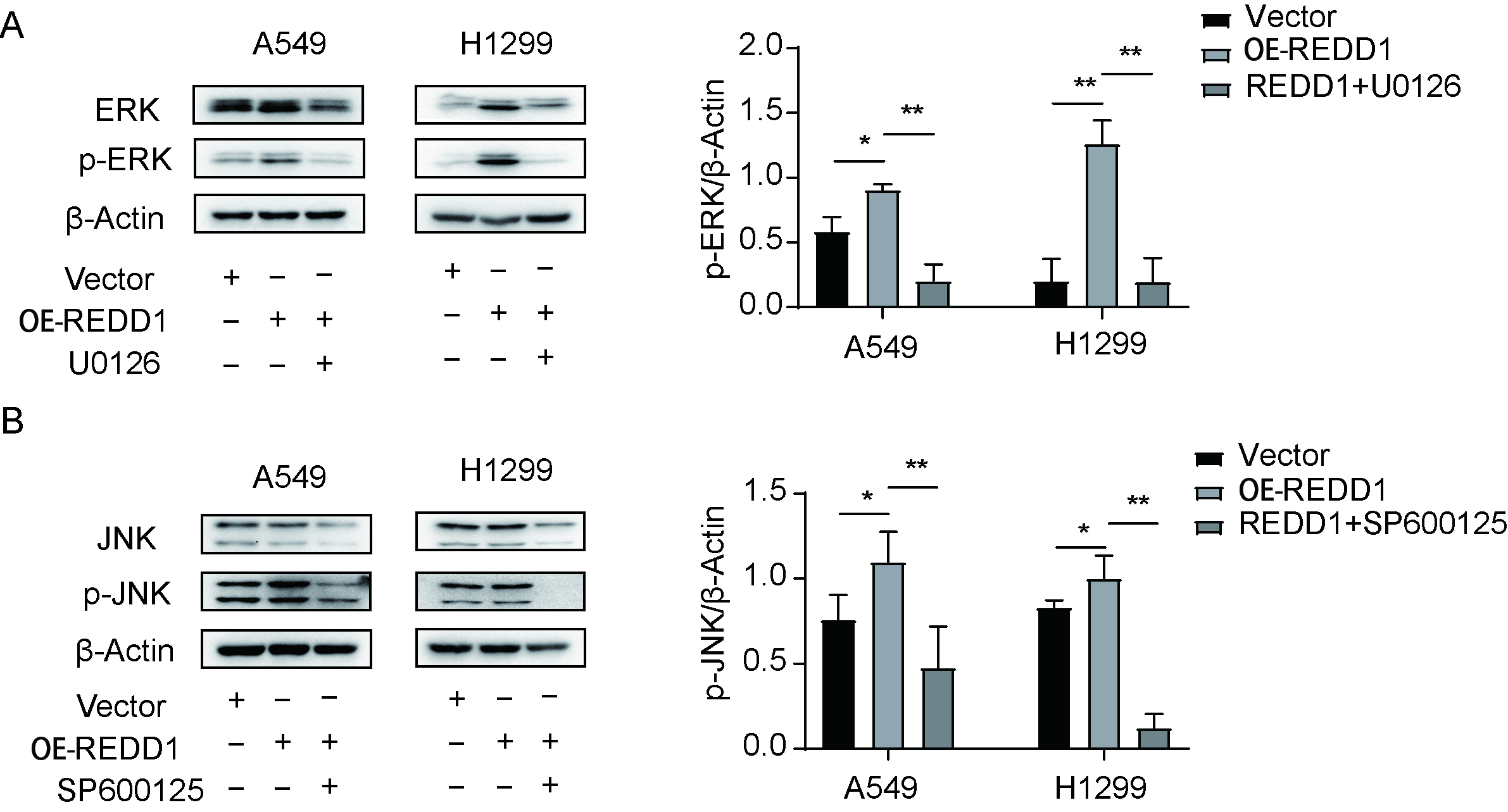

Supplement: Supplementary file 1 [file biomedicines-13-02918-s001.zip › Figure S6.tif]

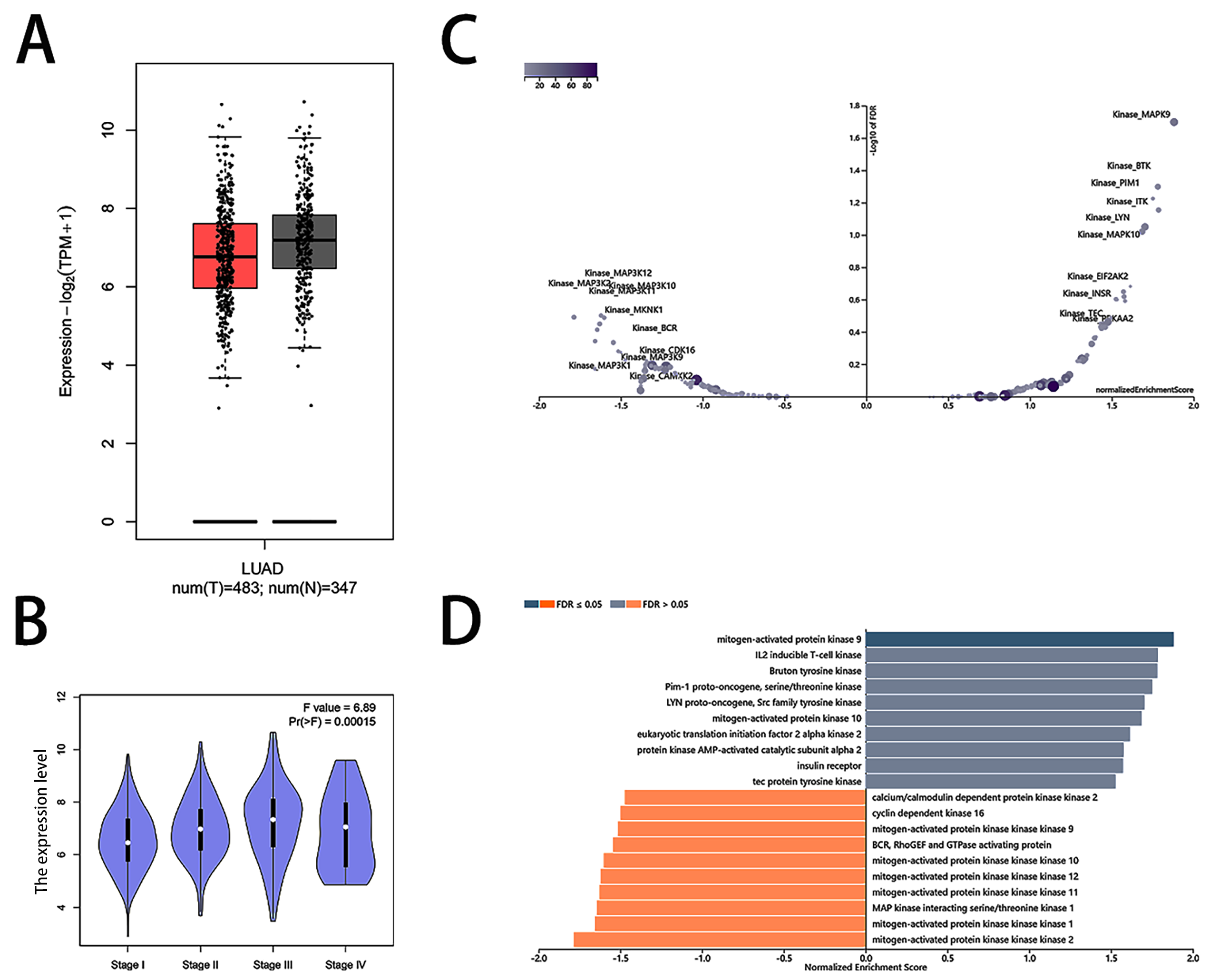

Supplement: Supplementary file 1 [file biomedicines-13-02918-s001.zip › Figure S7.tif]
